# Dental team barriers and enablers for the dental management of adults with severe obesity: a qualitative analysis

**DOI:** 10.1038/s41405-024-00264-x

**Published:** 2024-11-02

**Authors:** Zanab Malik, Kate A. McBride, Kathryn Williams, Deborah Cockrell, Clare E. Collins

**Affiliations:** 1 School of Health Sciences, College of Health, Medicine and Wellbeing, The University of Newcastle, Callaghan, NSW Australia; 2 Oral Health Services, Central Coast Local Health District, Gosford, NSW Australia; 3 Translational Health Research Institute, Western Sydney University, Penrith, NSW Australia; 4 School of Medicine, Western Sydney University, Penrith, NSW Australia; 5 Nepean Blue Mountains Family Metabolic Health Service, Nepean Hospital, Kingswood, NSW Australia; 6 Charles Perkins Centre-Nepean, The University of Sydney, Sydney, NSW Australia; 7 Food and Nutrition Research Program, Hunter Medical Research Institute, New Lambton Heights, NSW Australia

**Keywords:** Dentistry, Special care dentistry

## Abstract

**Background:**

Broad challenges regarding the dental management of people with severe obesity experienced by general dentists have been minimally explored. The perspectives of the dental team regarding these multifaceted issues are currently unknown and they potentially impede the delivery of optimal dental care to this population and contribute to poor oral and general health.

**Aims:**

Our qualitative study aimed to identify and explore barriers and enablers in the dental management of adults with severe obesity among dental professionals and support staff in Australia.

**Methods:**

Focus groups and semi-structured interviews (*n* = 34 participants) were conducted with dental professionals (*n* = 23) and support staff (*n* = 11). Recordings were transcribed verbatim and synthesised using thematic, inductive analysis.

**Results:**

Multiple barriers to adequate provision of dental care for people living with severe obesity in both general and specialist dental settings were identified. Key themes emerged related to the clinical challenges reported by participants in providing dental management for people living with severe obesity, appropriateness of existing bariatric dental service provision and safety of care. Enablers to access were identified, including increased availability of bariatric dental chairs, environmental modifications, education of both patients and the entire dental team and for guideline development.

**Conclusion:**

The current study explored multiple barriers to optimal dental management of people living with severe obesity in both general and specialist dental settings. Enablers should be used to inform future practice. The optimisation of existing bariatric dental service provision requires urgent review with solutions guided by systemic change. Study findings suggest a review of current health systems, economics, access barriers, policies and procedures and education and training beyond the individual level are needed. Future directions to improve the dental management of people living with severe obesity are proposed.

## Introduction

Global obesity prevalence is rising rapidly and is a major public health concern due to the increasing demand on the healthcare system [1]. Clinical obesity is a chronic relapsing progressive disease process, with this paper focussing on people living with severe obesity (those with a body mass index (BMI) of ≥40, or ≥35 with associated obesity-related health conditions) [2, 3]. Given the health impacts of obesity, and in particular severe obesity, it is imperative that people living at the severe end of the spectrum have appropriate access to all health service types, including dental services. This is important as obesity-related comorbidities often complicate dental management with some studies reporting poor oral health in people living with obesity compared with the wider population without obesity [4–6]. A study of 81 adults living with clinically severe obesity from a public hospital-based obesity service in New South Wales, Australia, found dental service utilization was poor, with more than half of the cohort (61.7%) reporting unfavourable visiting patterns [7]; including dental attendance only for a problem, rather than for preventative care. This was more than twice the unfavourable visiting patterns reported by 22% adults in the National Study of Adult Oral Health 2017–2018 in Australia [8]. Given obesity is a global issue, these findings are also of increasing relevance internationally and consistent with international evidence of weight stigma experienced by people living with obesity, resulting in avoidance of healthcare services in general [9]. Qualitative research exploring the perspectives of people living with clinically severe obesity has revealed several barriers to accessing dental services [10]. These include feeling disempowerment to act to improve their oral health, experiencing weight-related stigma and discrimination, unpredictability of the dental environment and a lack of tailored services [10].

In Australia, people living with obesity receive oral healthcare services in public and private general dental settings. However, people living with severe obesity, specifically those with very high body weights, necessitating the use of bariatric dental chairs, are frequently referred to public dental specialists in Special Needs Dentistry (SND) for comprehensive dental management [11, 12]. Bariatric chairs are indicated for use when the safe dental chair working limits are exceeded and are predominantly located within tertiary dental hospitals within SND departments [11]. The experiences and challenges of general dentists managing people living with severe obesity have been minimally explored and may be of increased relevance internationally in contexts without Special Needs Dentistry services. Qualitative research in the United Kingdom (UK) involving a group of twelve general dentists reported barriers such as caution with weight discussions and challenges in identifying weight and organising appropriate care [13]. A comprehensive literature review has failed to identify any existing literature exploring perspectives of the entire dental team, including both clinicians and support staff, in relation to the dental management of people living with severe obesity in Australia. Insights from dental specialists in SND are also currently unknown. These insights are key given their prominent role in current referral pathways.

This qualitative study aimed to identify and explore the barriers and enablers for dental management of adults living with severe obesity from the perspective of the dental team, including both dental professionals and support staff.

## Materials and methods

This study received ethics approval from the Central Coast Local Health District (CCLHD) Human Research Office for this Human Research Ethics Committee Exempt and Low/Negligible Risk research project (number 1122-101C). Numerous participants were invited to take part including dental professionals (registered general dentists, oral health therapists) and support staff (dental assistants, dental receptionists) working in private and public regional practices in New South Wales, and dental specialists in SND who were registered to work across Australia. Recruitment was via email to all public-based employees within CCLHD Oral Health Services and to registered dental professionals via the local mailing list from the Australian Dental Association. Recruitment for SND specialist participants was via email to the Australian and New Zealand Society of Special Needs Dentistry membership to include the 26 registered Australian specialist members at time of recruitment. The recruitment email included a focus group invitation letter and participant information sheet explaining the background to the research and research team.

All participants provided their informed consent to participate. A semi-structured interview schedule (Supplementary Fig. S1) was developed by a multidisciplinary project team including dental specialists in the field of SND and Oral Surgery, an academic nutritionist, and an endocrinologist obesity specialist. The focus group interview schedule was piloted with the first two consenting participants who were clinicians and who were agreeable to participate in semi-structured interviews which better accommodated their availability. The interview schedule was deemed appropriate for use with both clinicians and support staff and then only focus groups were scheduled with the remaining consenting participants. These participants were randomly allocated into focus groups with people of the same professional role. They were asked to sign a focus group confidentiality agreement form, to ensure privacy of discussions was maintained and encourage participation. Focus groups were either carried out via an online platform (Microsoft Teams™) or in person in a neutral non-clinical location.

Data were collected between March and May 2023. Focus groups were conducted by ZM, who is a female dental specialist in SND and was working part-time (2 days/week) in CCLHD at time of data collection. ZM has experience in qualitative data collection and the discussion topic and has clinical experience in the dental management of people living with severe obesity. A female research assistant (KK), with a research background in nutrition and qualified as an overseas-trained dental professional, was present during each of the focus groups. KK moderated the focus groups to ensure they were transparently conducted and assisted in reducing any bias with participants who were working or knew ZM prior to participation. Most interviewed participants were working at different sites to ZM within the public oral health service and unaware of the research prior to participation. All interviewed SND specialist participants were working at different sites to ZM, including interstate in Victoria, Queensland and South Australia. Non-SND specialist participants were unaware of the role of SND in managing people living with severe obesity prior to the research commencement. Field notes were made during and after each focus group by both ZM and KK. Data collection ceased once data saturation was reached, meaning when sufficient information had been obtained to replicate the study and no new further information was being collected [14].

Focus groups and the two semi-structured interview recordings were transcribed verbatim using transcription software Trint™ 2022, Trint Limited, United Kingdom (UK). Participants were offered the opportunity to review the transcripts, however no requests were made for review by participants. A thematic (inductive) analysis approach was undertaken to interpret the data and sort into themes and subthemes [15]. Following independent coding of two transcripts (10% of the data) by two researchers (ZM, KK), an initial coding framework was developed, with consensus checking conducted with researcher KM. Coding was performed using Quirkos 2.5.3 qualitative analysis software [16].

## Results

Thirty-four participants were recruited across clinician and support staff groups (Table 1). Five focus groups of between 2–3 participants each were conducted with support staff, including dental assistants (*n* = 9) and reception staff (*n* = 2). Seven focus groups of between 2–3 participants were carried out with clinicians, including general dentists (*n* = 8), oral health therapists (*n* = 5) and SND specialists (*n* = 8).

**Table 1:** Demographics of participants (*N* = 34) by gender, role, employment type and lived experience with obesity.

Participant characteristics	*N* (%)
Sex	
Female	27 (79.4)
Male	7 (20.6)
Role	
General dentist	10 (29.4)
Oral health therapist	5 (14.7)
Specialist special needs dentistry	8 (23.5)
Dental assistant	9 (26.5)
Dental receptionist	2 (5.9)
Employment type	
Public	23 (67.6)
Private	3 (8.8)
Mixed	8 (23.5)
Self-reported lived experience with obesity	
Yes	10 (29.4)

Three key themes relating to clinical challenges reported by participants in providing dental management for people living with severe obesity, the inappropriateness of current bariatric dental service delivery and issues around safety of dental care, were identified. Example excerpts relating to each theme and subthemes are presented in Tables 2–4. Excerpts reflecting enablers for the dental management of people living with severe obesity are presented in Table 5.

**Table 2:** Excerpts related to clinical challenges in the provision of dental care for people living with severe obesity by clinicians and support staff.

Emerging theme	Excerpt number	Excerpt
Ergonomics and positioning	2.1	'The one thing that you do get with the DOs and the DAs have it as well is during a procedure with a morbidly obese patient is getting in the right position and then they finish it and then backs really hurting, or their neck or their shoulders. Same when you’ve been in theatre and dealing with an obese person and yeah strange angles' *(Dental assistant)*
	2.2	'sometimes I find it really difficult to work with to and to actually position yourself correctly and things like that, and it is really difficult. Retracting cheeks, tongues, everything like that is really, really difficult.' *(Dental assistant)*
	2.3	'I sometimes find it quite hard with positioning to get into their mouth, especially if it’s like at the at the back of their mouth, like their back teeth. You kind of feel like you’re actually on top of them. And I try not touch them because you feel bad…it’s kind of like you’re just on the chest area…I don’t want them to see me avoiding it. But at the same time, I don’t want to just be like resting on them because I feel like that’s a bit insensitive as well… And actually physically leaning on them, It’s…I mean, some people don’t mind but it can be confronting' *(Dental assistant)*
Treatment provision barriers—time, transfers, dental chair failures, appointment scheduling	2.4	'we have to take more frequent kind of stops just to help with breathing and (they) couldn’t be like reclined fully in the dental chair as well…as it was too uncomfortable…as well really tight cheeks. So it was really hard to get access towards molar regions…It is difficult… having frequent stops obviously is individualised to the patient, but it is a little bit difficult to try to get the treatment done in the time frame' *(General dentist)*
	2.5	'getting them in and out of the chair…if they’re really obese, the chair doesn’t move as well. And there’s been times where the chair got stuck' *(General dentist)*
	2.6	'It’s quite difficult to arrange, so I think it took me a good maybe week or might have even been close to two to arrange this appointment to occur for this lady' *(Dental receptionist)*
Burden of dental disease	2.7	'they haven’t been able to go to the dentist or the general dentist, so they’ve neglected their oral healthcare for so long that by the time they present to myself that they’re in a state that could have easily been prevented had they come a bit sooner' *(SND specialist)*
Challenges in alternate dental settings—RACFs, theatre environments	2.8	'obesity is a huge headache when we take patients into theatre, the anaesthetic risks really escalate quite quickly for the obese patient and once again, certainly having a morbidly obese patient brings logistic challenges in the theatre for OH&S…can they get into a day stay unit or do they need to be admitted? I think the logistics of treating patients are becoming more onerous and this hospital in a state of what is it? 6 million people have got one bariatric chair in the whole hospital…so we’ve really got to identify that there is a need.' *(SND specialist)*
	2.9	'wheelchairs being large and not being accessible to, you know, doors, doorways, etc. So similar experience in residential aged care facilities where they are confined to large princess chairs and to make access a bit more convenient…they often get residents needing dental care to the hairdresser’s room or to another office where they all can come instead of us having to pack and unpack the mobile dental unit. But again, if they can’t get through the door of the hairdresser or the office room, then again we have to make that additional effort to go to their rooms where they are there. So that’s an additional barrier'*(SND specialist)*
Weight assessment, discussions or triaging challenges	2.10	'we’re told that we should ask questions in regards to weight to determine if they need a certain room set up, etc… I find that very difficult to ask personally over the phone, especially if you don’t have a rapport with that person' *(Dental receptionist)*
	2.11	'once…because they were really overweight…I had to check that the chair was suitable for this patient. And oh my God, I was like so nervous it probably took me like 5 min of standing outside to be like how do I…I talked myself into…I just feel uncomfortable asking “How much do you weigh?“' *(General dentist)*

**Table 3:** Excerpts related to the inappropriateness of current bariatric dental service delivery.

Emerging theme	Excerpt number	Excerpt
Inappropriate referrals	3.1	'We can have a laugh now at some of the referrals, because I think one of the referrals I received at when I was at the Oral Health Centre was someone, you know, considered a bariatric patient and therefore referred to the oral health centre. And then when I actually saw the patient, the person was actually edentulous and all the work had been done and (I was) thinking what a waste. So I had to definitely, I mean, call up the person who had referred and talk to them saying you’ve done all the work and the person is actually edentulous. I mean, why can’t you do pros(thodontic) work in the chair or wherever the person attends to. So I think that would be the classic wrong or inappropriate referral' *(SND specialist)*
Specialist vs general settings for bariatric dental care	3.2	'it starts to raise the philosophical question about is that where the care of people of patients with obesity needs to be? within specialized services or why shouldn’t they be able to access more general services…is this really a reliance that you know when you get to a certain weight or a certain BMI all of a sudden you can no longer receive any aspect of general dental care that you can’t go to a private practice, you can’t go to a community dental clinic, to receive the care that you need and automatically you need to be referred to a specialist unit. And then to sit on a, you know, extended wait list to be able to even get general aspects of your care' *(SND specialist)*
	3.3	'well in public I’ve experienced it a lot more. In private, I didn’t really experience obesity. I didn’t really see a lot of patients that way because they didn’t have bariatric chair so that wasn’t even a question' *(Oral health therapist)*
Inadequate facilities in specialist SND departments	3.4	'A gentleman that was referred from (a general dental clinic), he had an emergency extraction by sitting across three chairs in the waiting room after hours. And then he was referred to us because we are a specialty unit and because he had mobility issues, he couldn’t physically get into the bariatric room. The door wasn’t able to open wide enough to accommodate his mobility device!' *(SND specialist)*
	3.5	'As a specialty…maybe we’re an area that is more equipped to be able to manage these patients than elsewhere? I think that that’s a hard generalization for us to make cause often even within our specialized units is that we don’t necessarily have the facilities, the equipment, you know the access that’s necessary' *(SND specialist)*
	3.6	'And even our own specialty special needs department has a broken bariatric chair that is very difficult to fix because it’s from the UK. And so now we need to use the IV sedation one…the lack of support services or equipment makes it difficult for us to provide good service…we need to e-mail the other department to see if that chair is free, and then we need to bring all of our dental equipment down into another department and considering we’re the referral service for all bariatric patients, I feel like it just really doesn’t do them justice' *(SND specialist)*
Rationale for referrals or lack of awareness of referral pathways	3.7	'I guess with dental treatment I would be looking to maybe contact Westmead or Sydney Dental Hospital who have yeah more equipment and services available and specialists available and maybe going that way or speaking with my line managers and finding out what their suggestion would be if our equipment wasn’t suitable' *(General dentist)*
	3.8	'I often forget that that could be something that I would refer to a Special Needs Dentist, I often think a Special Needs Dentist might need to manage our patients that have physical disabilities, intellectual disabilities, and I forget that that is something that they might manage. Often I just think I’ll just book that with any dentist as long as it’s in that chair. And I forget that there’s someone who may do that better…Because you don’t think of it as a disability…I guess they do have lots of other medical conditions sometimes as well, that do link in. So having a more complex medical issue, having a specialist is important' *(Oral health therapist)*
	3.9	'if there was some reason why I couldn’t proceed as normal or provide optimal care I’d refer if it’s really complex and I’m worried about encountering something that will be out of my scope, if they’re at risk of a medical emergency or something in the chair due to their weight and medical conditions…But if I had a patient who was overweight and I was able to provide the care as I normally would, I wouldn’t automatically think to refer them to a special needs dentist if they are happy to see a general practitioner' (*General dentist)*
	3.10	'But in private practice, definitely a bit more challenging because… if the patient was over that (chair weight), it would be hard to…I wouldn’t know where to refer the patient if they weren’t eligible for public treatment…to be honest' (*General dentist)*

**Table 4:** Excerpts related to safety of dental care for people living with severe obesity.

Emerging theme	Excerpt number	Excerpt
Medical risk with dental procedures for those living with severe obesity	4.1	'We’ve been fortunate in not having any encounters, but …when we used to have the DIACO platform (bariatric chair), I used to always worry about what would happen if patients needed, advanced life support (laughs) because the bariatric platform really does not support any… compressions or weight… you know, if we were going to do CPR and to quickly lower them or to lie them on their sides like that? …we just really haven’t had training as to what we would do if the situation arose. But the IV Sedation Department, which has the other bariatric chair I think is a safer place for our patients, if they did need a emergency management because it’s…, located with all the equipment that’s for…CPR and for yeah life support. And there is often other sedationists around who could help out and nurses who are all trained in emergency management. And the chair itself is a lot more… anchored to the floor…it’s a lot more sturdy too to help us manage' *(SND specialist)*
	4.2	'I feel fairly equipped. I mean, I don’t know if it would differ. I mean, the only thing would be like the airway, but I don’t think my management would differ in any medical emergency' *(General dentist)*
Treatment planning modifications for patient safety	4.3	'I guess you know other comorbidities is one of the big factors to consider, but I think it’s the airway management which is the main risk for these patients. So sometimes it might be that their general anaesthetic risk is too high that we have to consider potentially other options to provide treatment for these patients. But you know such as IV sedation or inhalation sedation or something like that instead, but even that in itself will have other risks, and that’s where… really being involved with the patient’s medical team and actually other specialists involved to provide the safe planning for these patients is really, really important. So It’s not something that you can just go, Yep, that’s fine straight to general anaesthetic. All those risk factors that we have to keep into consideration for these patients' *(SND specialist)*
	4.4	'I guess for GA a lot more of them need overnight admissions due to higher risk' *(SND specialist)*
	4.5	'to ensure that they are medically optimised before we undergo surgical procedures, for example, because that will affect wound healing…we refer them back to their GP and form that link to you know manage some of those medical comorbidities before we plan for sort of surgical procedure' *(SND specialist)*
Physical environment accommodations	4.6	'It’s not just the surgery themselves, it’s the waiting room, it’s our toilet facilities. I don’t know if one of the toilets outside is, but inside we have a wheelchair accessible toilet. I don’t think the others are…in the waiting room, they would struggle to sit on the seats that we have and are those seats strong enough for somebody who’s morbidly obese to sit on?…Just thinking about our building – the parking isn’t ideal for many disabled patients/obese patients at once. And then the ramp getting up to the clinic would be quite tricky for a lot of obese patients. And a lot of our doorways, they are not very wide at all. We need big doorways' *(Oral health therapist)*
Private vs public and safety considerations	4.7	'in private practice that I’m working in at the moment…things to think about is how close to the nearest hospital … particularly for patients that maybe…there’s extra precautions or you know, it might be more difficult for them to get into the ambulance … I feel in my current setting that that’s something that I would not be very well equipped to deal with…and the other issue we talked about before, the bariatric chairs at least that are close to me are all in public settings as well' *(SND specialist)*
Weight assessment for safe dental treatment	4.8	'main thing that I can think of is just when it did come to their weight specifically, knowing whether it would be safe to treat them in the, you know, in a certain chair' *(General dentist)*

**Table 5:** Excerpts related to enablers for the dental management of people living with severe obesity.

Emerging theme	Excerpt number	Excerpt
Physical environment/appropriateness of available facilities in all dental settings	5.1	'we have Yerin, that’s a culturally appropriate place for our indigenous patients to be seen. So why don’t we have a clinic that’s more appropriate for our management of our patients with obesity?' *(Oral health therapist)*
	5.2	'we’ve got the ramp that leads into the clinic…quite a wide waiting room. We’ve got wide hallways leading through to the from the waiting area to the patient room. And then the room itself is very, quite spacious, which is extremely important…and that’s the reason that we can’t treat in the other rooms because there’s just not sufficient space in half of the rooms here. So that’s 100% integral for the patient, but also for transportation using the hoist, wheelchair…and then obviously just having the right chair to be able to manage' *(General dentist)*
	5.3	'wheelchair tipper, I think is a great facility to have. And very beneficial not just for patients with obesity, but for patients with physical disabilities as well that can’t transfer to the dental chair' *(SND specialist)*
	5.4	'…having individual cubicles. I think from a patient point of view having that dignity and respect to kind of … have their own area, not have those big open cubicles that you sometimes see in the general clinics. I think that’s something (for) … future planning for a clinic' *(SND specialist)*
	5.5	'I think we’re quite equipped to managing patients with obesity because we do have the bariatric chair and then we also have access to special needs dental specialist' *(General dentist)*
	5.6	'I think in terms of the private sector, you can’t exclude somebody just because they’re obese. There has to be accommodation in some of the private specialist practices to identify these urgencies' *(SND specialist)*
	5.7	'having a bariatric chair is not the answer…I think also there needs to be some training of staff that are actually seeing patients there as well because it’s reasonably high frequency of injuries to staff. They don’t receive any formal training. They just told to get on with it… And you know you don’t want to get to a point where they say we don’t want to see those patients because I got injured last time. That’s a real concern' *(General dentist)*
Education of patients and the entire dental team and for guideline development	5.8	'there’s a need to advocate more broadly within our profession and outside our profession…for example, in the medical sphere, raising awareness … making them aware of the referral pathways …. and patients that we see' *(SND specialist)*
	5.9	'I think if you’re going to have a guideline about the dental management of obese patients, you’d …want kind of the practical access discussion that we’ve had and the medical management. … such as protocols when it comes to medical emergencies… so that all clinical settings just have a really effective way to safely manage …obese patients' *(General dentist)*
	5.10	'physical environment, physical access, OHS, safety, that’s one. I think the medical considerations of the consequences of obesity… and also the psychosocial elements… would be very helpful' *(SND specialist)*

### Clinical challenges related to the provision of dental care for people living with severe obesity

Clinical barriers in the provision of dental treatment for people living with severe obesity reported by clinicians and support staff, including work health and safety (WHS) issues, ability to optimally perform procedures, length of treatments and provision of appropriate care. Ergonomic concerns were reported (excerpt 2.1) regardless of whether a conventional dental chair or bariatric dental chair was used. This impacted on WHS, with participants in this study reporting pain after undertaking procedures on people living with severe obesity due to poor positioning. Dental assistant participants shared their own unique barriers, including challenges around retraction of soft tissues and suctioning, which influenced their confidence in assisting in procedures involving patients living with severe obesity (excerpt 2.2). They also shared their embarrassment of invading patients’ physical spaces to carry out these tasks (excerpt 2.3).

For clinician participants, elongated treatment times occurred due to the additional challenges with frequent breaks required. The experience of the dental chair not working or 'getting stuck' was shared by many participants (excerpt 2.5). Logistic difficulty in scheduling appointments was also reported by several support staff participants, particularly dental receptionists. This was due to additional time required to cater for specific needs such as organisational delays with ambulance or community transport, awaiting specific equipment, or bariatric dental chair availabilities (excerpt 2.6). Other barriers to the provision of optimal care were patient factors, including patients presenting with anxiety and/or attending for predominantly emergency visits, with high burden of dental disease (excerpt 2.7), and challenges with assessing patient weight to determine safe dental chair selection (excerpts 2.10,2.11).

Barriers were also identified when delivering dental care to people living with severe obesity in alternate dental settings, such as residential aged care facilities (RACFs) or theatre environments. SND specialists highlighted the challenges of logistical planning for dental management under general anaesthesia due to higher anaesthetic risks for patients living with severe obesity (excerpt 2.8). In domiciliary service provision, for example in RACFs, rapport building was hindered by the limited interaction possible with patients and additional time was required to transport portable dental equipment when people living with severe obesity were unable to mobilise (excerpt 2.9).

### Inappropriateness of current bariatric dental service delivery

Another key theme was the inappropriateness of current bariatric dental service delivery in Australia, which was reflected in existing referral processes and existing pathways to treatment and facilities, including the reason for referrals. A major subtheme for SND specialist participants were referrals, specifically inappropriate referrals. This included ‘automatic’ referral of people living with severe obesity who exceeded the conventional dental chair weights to SND specialists to access use of bariatric dental chairs. As a result, the dental care of people living with severe obesity may have been delayed due to lengthy specialist waiting lists when the treatment needs were within the scope of a general dental service. This was highlighted in the example of the edentulous patient requiring denture construction, who was referred on the basis of their weight (excerpt 3.1). However, the dental treatment needs were not requiring SND specialist management and could have been carried out by any general dentist or dental prosthetist if the patient had access to a bariatric dental chair.

The ethical or moral dilemma of the specialist referral in itself was perceived as being stigmatising, in circumstances when appropriate care for people living with severe obesity could be provided in private or public general dental clinics if they were better resourced (excerpts 3.1, 3.2). A generalised lack of bariatric facilities in private dental settings was reported, leading to lack of exposure to this type of patient for private-based clinicians (excerpt 3.3). SND specialists contributed further insights into inadequate facilities, even within specialist SND departments, including examples such as lacking suitable toilets or equipment for people living with severe obesity (excerpts 3.4, 3.5, 3.6).

Referrals were deemed to be more appropriate and initiated in settings where equipment facilities were lacking, such as in the absence of a bariatric dental chair, in both private and public general dental settings (excerpt 3.7). Where bariatric dental chairs were available in a public setting, dental management was able to be carried out by general dentists and referral for specialist management in SND departments was often not considered by general dentists or oral health therapists (excerpts 3.8, 3.9). However, if the patient wasn’t eligible for public dental services and required a bariatric dental chair for management, referral pathways to SND were then unknown (excerpt 3.10).

### Safety of dental care for people living with severe obesity

Safety of dental service delivery was a key theme and challenge discussed by participants. This centred around medical risks and impacts on dental treatment planning, including aspects such as the physical environment and weight assessment. Predominantly SND specialist participants acknowledged the medical risk around procedures (excerpt 4.1) whereas in contrast, most of the other clinicians and support staff did not appreciate these risks (excerpt 4.2). Several felt they would be equipped for medical emergencies, though others admitted they did not know how emergency management would differ for the patient living with severe obesity. This suggested a lack of experience or knowledge of the complexities that people living with severe obesity may bring to the general dental setting.

SND specialist participants were able to provide insights into treatment planning considerations under differing treatment modalities, such as intravenous sedation or general anaesthesia in greater detail and raise considerations of safety and risk (excerpts 4.3, 4.4). Other clinician participants raised the dilemma for alternative treatment options when patients were deemed not suitable for general anaesthesia if this was their initially planned treatment modality. The need for patients to have improved stability in their medical comorbidities ahead of planned surgical dental interventions for improved patient outcomes and safety was also reported by SND specialist participants (excerpt 4.5).

Safety of dental care provision for people living with severe obesity was also related to the surrounding physical environments needed to meet their needs, such as having adequate facilities in the dental clinic (excerpt 4.6). Some participants relayed the differing environments available in public versus private settings and how weight assessments could impact on safety of dental care (excerpts 4.7, 4.8).

### Enablers for the dental management of people living with severe obesity

Participants provided numerous suggestions for enablers to improve the dental management of people living with severe obesity. This included facility-based recommendations, such as increased availability of bariatric dental chairs and other environmental modifications to ensure appropriateness of available facilities across both public and private settings (excerpts 5.1–5.6). Participants also emphasised the need for regular training in conjunction with bariatric chair usage to prevent injury to themselves, as well as regular breaks during appointments (excerpt 5.7).

Enablers to access suggested by SND specialists predominantly centred around education of their local teams, the wider profession and medical colleagues regarding the category of bariatric patients that ideally should be referred for specialist management, with characteristics not based on weight assessment alone (excerpt 5.8). Improving the appropriateness of referrals was perceived as being a primary measure to improve access. There was a desire to have guidelines and training for both clinicians and support staff in obesity dental management (excerpts 5.9, 5.10). Guidelines were recommended to cover the broad medical, physical and psychosocial aspects relevant to dental care. There was some confusion among participants around the weight limits of bariatric dental chairs, or of the general dental chairs within facilities, with most participants reporting that they were unsure of these. This reflects the need for further education and training before weight discussions can be initiated with patients.

## Discussion

This study found multiple barriers and enablers to providing dental management for people living with severe obesity, from the perspective of clinicians and support staff in the Australian context. Barriers in this study related to communication, access, resources, work health and safety and policies and procedures. There was consensus on the challenges and barriers from participants for the provision of dental care for people living with severe obesity, particularly when requiring management in a bariatric dental chair. The barriers in the Australian context were similar to those reported by UK-based dentists with regards to difficulty broaching the topic of weight, equipment and safety and problematic referral pathways [13]. Differing barriers were highlighted from SND specialist in this study compared with general clinician participants particularly around safety of dental care with a focus on medical risk and treatment modalities, likely due to the increased complexity of patient cases they manage. This study also provided detailed insight into barriers to dental management for people living with severe obesity in differing settings, such as theatres or RACFs from SND specialist participants. Clinician and support staff participants raised concerns relating to WHS and patient safety, through their experiences of difficulty performing their work, airway and medical emergency risks which were previously unknown. Whilst the literature has reported barriers relating to WHS, this has been based on expert opinion [11, 12, 17, 18]. This study uniquely provided insights into specific aspects of clinical management that are of concern relating to WHS and patient safety from a clinical perspective, allowing for practical strategies for the dental team to now be developed to overcome these barriers. These may include increased appointment times, strategic breaks, workplace stretching to reduce the risk of musculoskeletal strain, equipment and training to ensure optimum ergonomics when managing patients living with severe obesity.

SND specialist participants expressed concern regarding existing referral pathways worsening current access barriers due to specialist waiting times. This is exacerbated by limited placement of bariatric dental chairs in public specialist SND departments in several states in Australia. This may be an Australian-specific issue but is likely to be of global relevance given the increasing worldwide obesity rates. Urgent attention is required given the moral and ethical questions it raises as it discriminates against patients due to their weight, particularly in settings where inappropriate referrals are being made based on weight alone. Additionally, specialist SND participants clearly identified that specialist dental facilities are also not well equipped and barriers to adequate care of patients with severe obesity still persist. The poor awareness, use and availability of existing referral pathways for specialist SND services across Australia remains problematic. Furthermore, inappropriate referrals and confusion around the role of SND in bariatric dentistry make professional advocacy challenging despite the additional knowledge and training of SND specialists, making them well-placed to provide the education that is currently lacking.

Increased education and the development of clinical practice guidelines have been recommended in the literature as an enabler to access for the medical management of obesity [19]. This was also a key finding of this study, which further emphasised the need of practicing clinicians and support staff for clinical dental practice guidelines in Australia, which had previously not been identified.

Our participants acknowledged that improved access to bariatric dental facilities is required, beyond just increased availability of bariatric dental chairs in both public and private general dental settings. Purpose-built specialised bariatric dental facilities are urgently needed, underpinned by a focus on enablers to access and demand for services. Overall, the results of our study suggest systemic and physical environment changes need not be limited to SND specialist dental settings and that more needs to be done to improve access to dental care for people living with severe obesity. As such, to address the barriers reported by participants, interventions are required beyond education alone which has been the current focus in the literature relating to severe obesity in the dental setting [10]. There is a need to adopt an integrated, whole-system approach to overcome widespread access barriers, resourcing and to restructure existing referral pathways, policies and guidelines relating to occupational health and safety, education and training.

### Future directions and recommendations

Incentives for private-based dental practitioners or for new practices to make the physical infrastructure modifications to the clinical setting should be considered. Positive impacts of adequate services catering to people living with severe obesity have been observed in the medical sphere, where specialist multidisciplinary obesity medical services have had a significant impact on reducing acute hospital presentations for people living with severe obesity [20]. Despite these significant benefits, these services are both limited in number as well as under-resourced, indicating that the general healthcare setting in Australia is under- equipped to manage obesity [21]. Given the predictions for increasing prevalence of severe obesity in Australia, likely costs to the health system and disability-adjusted life years [22–25], adequate dental access and care for people living with severe obesity is imperative to prevent the reduction in quality of life associated with oral health problems [26].

Suggested future service revision could also include integration of dental services within existing multidisciplinary obesity services. Models of care for bariatric dental patients through government-funded subsidies similar to the now-defunct Medicare Chronic Disease Dental Scheme [27] may be another strategy for consideration. The narrative around obesity requires change to improve access to all healthcare services and is not unique to dentistry.

### Strengths and limitations

Limitations of this study include the predominantly female sample of non-specialist SND participants recruited from a single regional geographical region. The sample may therefore not have been representative of all dental clinicians and support staff. There may also have been sampling bias as participants with lived experience of obesity, or experience of managing patients living with obesity, may have been more willing to participate in the study. The data is also limited by the inherently subjective nature of the qualitative interview data which may have contributed to an under reporting of weight stigma experiences by participants.

However, the current study had some important strengths. The piloting of the interview schedule and semi-structuring of focus groups used in this study ensured sensitivity in relation to this topic and that questions would be interpreted correctly. The focus group methodology employed was advantageous to elicit broad exploration of the topic of barriers and enablers to dental management for those living with severe obesity [28]. The number of focus groups carried out was sufficiently high to have ensured key perspectives from both clinician and support staff groups. Another strength of this qualitative study was the inclusion of perspectives of various dental team members. The study also uniquely considered the perspectives of SND specialists nationally, given their prominent role in existing referral pathways across Australia and they were able to provide their unique insights from differing contexts in their respective states. To the authors’ knowledge, this is the first investigation of the perspectives of Australian clinicians and support staff in managing people living with severe obesity. Given the differing context and limited access to SND services within Australia compared with community clinic access in the UK, the unique specialist considerations provide previously unreported data.

## Conclusion

The current study explored numerous barriers for the dental management of people living with severe obesity in both general and SND specialist dental settings. Enablers should be used to inform future practice. The optimisation of existing bariatric dental service provision requires urgent review with solutions guided by systemic change. The findings of this study suggest review of current health systems, economics, access barriers, policies and procedures and education and training beyond the individual level. Future strategies to improve the dental management of people living with severe obesity are proposed.

## Supplementary information

SI Fig 1: Semi-structured focus group interview schedule

## Data Availability

The datasets generated and/or analysed during the current study are not publicly available due to the sensitive nature of focus group discussions but are available from the corresponding author on reasonable request.
