# Supplementary material for: Dental team barriers and enablers for the dental management of adults with severe obesity: a qualitative analysis
Source: BDJ Open. 2024 Nov 2;10:83. doi: 10.1038/s41405-024-00264-x (PMC11531496; doi:10.1038/s41405-024-00264-x)
Supplement: Supplementary file 1 — SI Fig 1: Semi-structured focus group interview schedule [file 41405_2024_264_MOESM1_ESM.pdf]

Obesity STIGMA-DENT STUDY Focus group questions – support staff

-Tell me about your experiences in interacting with adult patients with obesity in the workplace?

-Are you aware of any challenges that adult patients with obesity may experience in your place of work? How might you work around these?

-What is your understanding of obesity and what can be done about it? Does your workplace have any specific interventions or procedures to help with the dental management of adult patients with obesity?

Thanks for your time and participation.

Obesity STIGMA-DENT STUDY Focus group questions – clinicians

1.What is your understanding of adult obesity and what can be done about it? What do you feel is your role (if any) in the prevention or management of obesity?
SubQ1: How have you experienced asking adult patients questions around weight? How has the response been from patients in your opinion and does this influence your approach? Describe any differing experiences (if any) when managing adult compared with paediatric patients with obesity? Why do you believe this might be different?

SubQ2: Compared with your usual preventive oral healthcare advice (including diet and oral hygiene) are there any changes you would make to the preventive advice you would give to a patient with obesity? Do links between obesity and periodontal disease or caries form part of your preventive discussions with patients with obesity?

2. Thinking of when you (or your colleagues) have treated adult patients with obesity, describe your experiences, including any challenges or barriers to their dental management that you have encountered
SubQ1: What are your most important considerations in your dental management when managing adults with obesity including using different treatment modalities such as conscious sedation and general anaesthesia?
SubQ2: Describe any differences in your dental management or approach depending on the level of obesity patients present with. Describe any protocols you have in place?

SubQ3: Specify the kind of weight limits you think the dental chairs in your practice have? Describe any experiences with patients who have exceeded the safe dental chair working limits? How did you manage these situations?

Sub Q4: How have you (if ever) discussed referral of a patient who exceeds safe dental working limits of the dental chair where you work? Are you aware of the nearest bariatric dental chair

located near you and how far away is it from where you work? In your opinion, how are patients with clinically severe obesity who can't attend your dental practice or be managed in a conventional dental chair accessing their dental treatment especially in emergency dental situations like a facial swelling?

SubQ5: Describe when and why you might refer a patient with obesity for their dental management and to whom (if anyone) and what this referral pathway might involve?

What do you consider to be the role of the special needs dentist in dental management of people with clinically severe obesity (and if you are a special needs dentist what does this involve?)

Sub Q6: How equipped do you feel if a patient with obesity had a medical emergency in your dental practice?

3.What kind, if any, barriers might adults with obesity be facing in your workplace?

SubQ1: How do you feel your facility is equipped to manage adults with obesity and what kind of things could be improved? What elements have you considered in the design of your facility or do you feel are integral from when they arrive to when they leave?

Thanks for your time and participation.
